# Germicidal effect of intense pulsed light on *Pseudomonas aeruginosa* in food processing

**DOI:** 10.3389/fmicb.2023.1247364

**Published:** 2023-08-24

**Authors:** Jinglong Liang, Teng Yi Huang, Xuejie Li, Yan Gao

**Affiliations:** ^1^College of Light Industry and Food Technology, Zhongkai University of Agriculture and Engineering, Guangzhou, China; ^2^Guangdong Provincial Key Laboratory of Lingnan Specialty Food Science and Technology, Zhongkai University of Agriculture and Engineering, Guangzhou, China; ^3^Key Laboratory of Green Processing and Intelligent Manufacturing of Lingnan Specialty Food, Ministry of Agriculture, Zhongkai University of Agriculture and Engineering, Guangzhou, China; ^4^Department of Diagnostics, Second Affiliated Hospital of Shantou University Medical College, Shantou, China; ^5^Guangdong Province Key Laboratory for Green Processing of Natural Products and Product Safety, School of Food Science and Engineering, Engineering Research Center of Starch and Vegetable Protein Processing Ministry of Education, South China University of Technology, Guangzhou, China; ^6^Research Institute for Food Nutrition and Human Health, Guangzhou, China; ^7^Department of Genetics, Genomics, and Informatics, University of Tennessee Health Science Center, Memphis, TN, United States

**Keywords:** intense pulsed light, *P. aeruginosa*, food processing, biofilm, planktonic bacteria

## Abstract

**Background:**

*Pseudomonas aeruginosa* (*P. aeruginosa*) can cause serious infections in many parts of the body and is also an underestimated foodborne pathogen. Intense pulsed light sterilization is recognized for its high sterilization efficiency, flexible and safe operation and ease of installation on production lines, which makes up for the shortcomings of several other physical sterilization technologies.

**Methods:**

This experiment studied the killing efficiency of different capacitances (650 μF, 470 μF, and 220 μF) of intense pulsed light on foodborne pathogenic microorganisms *P. aeruginosa* in the models of liquid food models, 96-well cell plates, and polycarbonate membrane models at room temperature (25°C) and refrigerated (4°C) environments to provide data to support the application of IPL sterilization devices in food processing.

**Results:**

The IPL was very effective in killing *P. aeruginosa* in the planktonic state as well as in the early and mature biofilm states, meeting target kill rates of 100%, 99.99%, and 94.33% for a given number of exposures. The biofilms formed in the polycarbonate membrane model and the 96-well plate model were more resistant to killing compared to the planktonic state. To achieve the same bactericidal effect, the number of flashes increased with decreasing capacitance.

**Conclusion:**

The bactericidal effect of IPL on *P. aeruginosa* was significantly influenced by the state of the bacterium. The larger the capacitance the higher the number of pulses and the better the sterilization effect on *P. aeruginosa*.

## 1. Introduction

Food items such as bottled water, milk, meat, vegetables, fruit, and seafood undergo a series of food processing processes before they are put on the market or retail shops for sale (Augustin et al., 2016). During processing, food products may be at risk of contamination by various pathogenic microorganisms, commonly including *P. aeruginosa*, *Escherichia coli* O157:H7, *Staphylococcus aureus*, *Salmonella*, *Vibrio parahaemolyticus*, *Listeria monocytogenes*, and fungi etc. (Joseph et al., 2001; Tompkin, 2002; Kadariya et al., 2014; Xu et al., 2021b; Liu et al., 2022a, 2023c). Foodborne pathogens can pose a serious public health risk to humans, and it is estimated that foodborne or waterborne diarrhea alone causes approximately 2.2 million deaths worldwide each year (Cisse, 2019). The USA federal government estimates that there are approximately 50 million cases of illness caused by foodborne pathogens each year (Gourama, 2020). Despite active and diligent efforts by governments to establish a range of food safety standards, cases of pathogenic bacteria being detected in food continue to occur.

*Pseudomonas aeruginosa* is a food-borne pathogenic gram-negative bacterium that is often detected in drinking water, dairy products, meat, etc. (Xu et al., 2019). *P. aeruginosa* is frequently transmitted via the consumption of bottled or barrel water, leading to detrimental effects on human health (Li et al., 2023a,b). The Chinese National Standard for Natural Mineral Water for Drinking Purposes (GB8537-2008) and the National Food Safety Standard for Packaged Drinking Water (GB19298-2014) specify that *P. aeruginosa* is “not detectable.” Because it can survive and proliferate in water, *P. aeruginosa* is easy to cause secondary contamination. *P. aeruginosa* is capable of causing otitis media, urethritis, endocarditis, gastroenteritis, pneumonia, and septicemia, whose main potential pathogenic factor is toxin A, in addition to lipopolysaccharide, protease, pili, leucocidin, and hemolysin, etc. (Woods and Iglewski, 1983). Further studies revealed that the expression of virulence factors of the opportunistic pathogen *P. aeruginosa* was regulated by the quorum sensing system, which consists of two pathways, *las* and *rhl* (Pesci et al., 1997; Smith and Iglewski, 2003). The emergence of multiple antibiotic-resistant strains has led to a dramatic increase in deaths from *P. aeruginosa* in hospitals, particularly in immunodeficient patients (Bodey et al., 1983; Bassetti et al., 2018). The main mechanisms of antibiotic resistance in *P. aeruginosa* include, synthesis of endocannabinoids and aminoglycosides, activation of the efflux pump and reduced cell membrane permeability (Lambert, 2002; Breidenstein et al., 2011).

*Pseudomonas aeruginosa* also has the distinctive feature of forming biofilm, which is thought to be one of the reasons for the pathogenicity and antibiotic resistance of the strain, for example, 75% of isolates from patients with ventilator-associated pneumonia exhibited strong biofilm-forming capabilities, and approximately 53% of these isolates exhibited multidrug resistance. Biofilms are sophisticated, articulated communities of bacteria that can adhere to the surfaces of medical equipment, household plumbing, factory equipment, food, etc. (Xu et al., 2021a, 2022). Extracellular polysaccharides, proteins, lipids, and eDNA are the most important components in the formation of the complex biofilm matrix, which provides a protective barrier for bacteria against harsh environments, antimicrobial agents and host immune effects (Liu et al., 2022b, 2023a,b; Wen et al., 2023). The biofilm cycle consists of three main stages, namely adsorption onto the surface of living or non-living media, biofilm maturation, planktonic cell dispersion. Tomatoes, lettuce, spinach, cabbage, and dairy products are also favourable sites for biofilm formation by *P. aeruginosa* (Xu et al., 2019). In addition to the various foods mentioned above, *P. aeruginosa* is able to form biofilm in nutrient-rich, moist environments, including pipes, sewers and floors (Meliani and Bensoltane, 2015).

IPL sterilization, a safe (mercury free), powerful, energy efficient, non-thermal sterilization technology, uses pulses of intense white light to cause inert gas lamps to emit light in the ultraviolet to infrared region similar to the sun’s spectrum but more intense to inhibit the growth and reproduction of microorganisms on the surface of food and packaging materials, on solid surfaces, in gases and in beverages. Germicidal UVC disrupts the pyrimidine dimer structure of bacteria (mainly thymine). The structurally altered dimer inhibits the formation of new DNA strands, thereby inactivating the microorganism. This is accompanied by the destruction of cell membranes, proteins and other macromolecules. Compared to non-pulsed or conventional UV sources, pulsed light has been shown to have a significantly higher germicidal effect, with one to several pulsed flashes killing microorganisms at a high level. The results of the study by Cheigh et al. (2013) showed that IPL could inactivate *Listeria monocytogenes* more effectively and promptly than conventional UVC treatment. The aim of this study was to evaluate the bactericidal effect of IPL on different states of *P. aeruginosa* during food processing, including planktonic bacteria in water or milk, biofilm on the inside of pipes or containers, or biofilm on food surfaces, at temperatures of 25°C and 4°C.

## 2. Materials and methods

### 2.1. Study design

**Figure 1:**
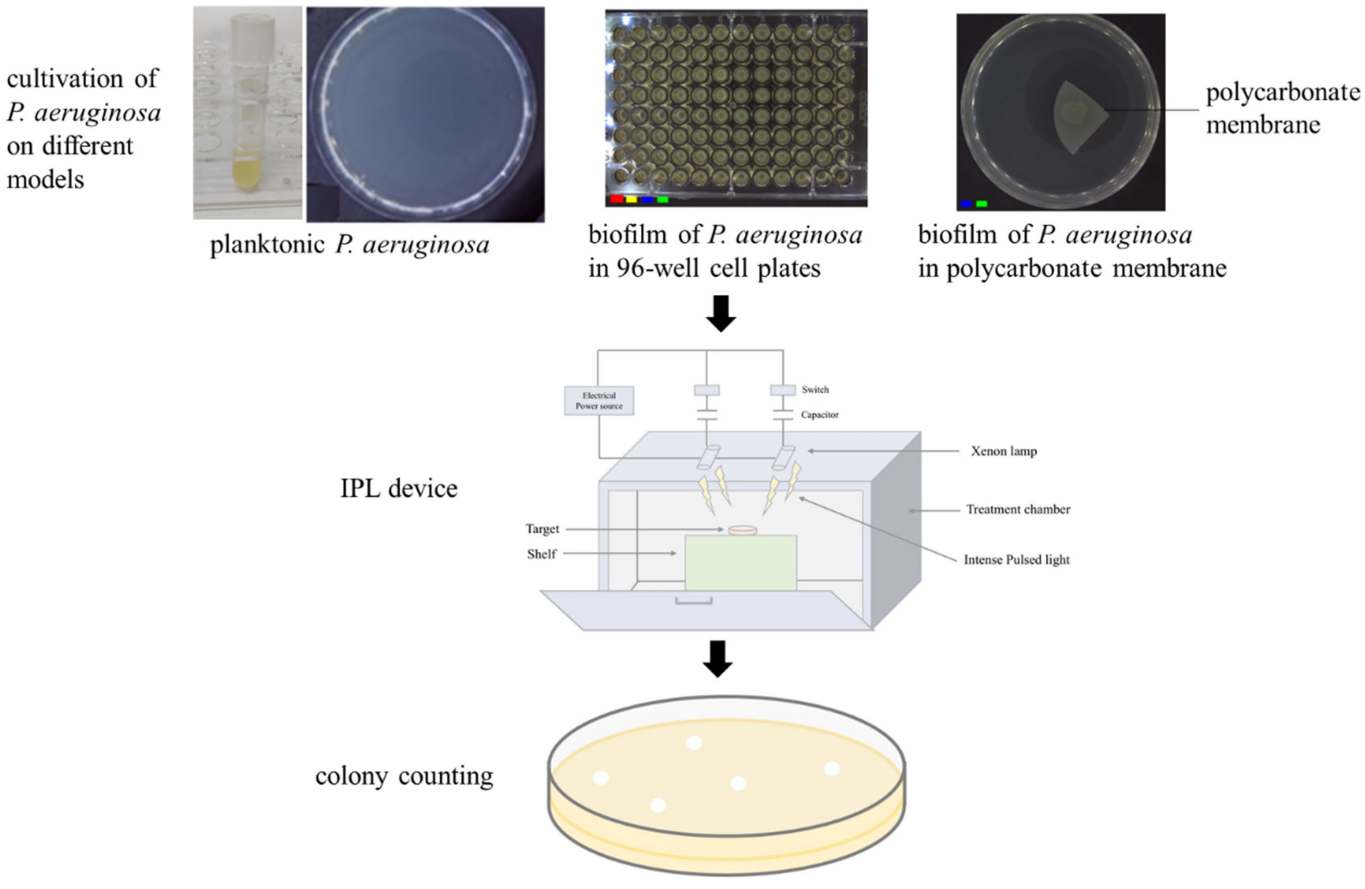
Schematic diagram of the IPL device and three models used in this study.

Since *P. aeruginosa* can cause secondary contamination through drinking water, it is very harmful to human health. In this study, to explore the inactivation effect of *P. aeruginosa* by IPL more comprehensively, we constructed three models: (I) simulating the growth of *P. aeruginosa* in liquid food such as drinking water by the planktonic growth state; (II) to mimic the formation of biofilms by *P. aeruginosa* within containers or pipelines, a 96-well cell plate biofilm model was employed to simulate the growth of the bacteria and the subsequent biofilm formation; (III) simulating the growth of *P. aeruginosa* on the surface of solid food with nutrient supply state by the colony biofilm (Figure 1), based on the growth characteristics of *P. aeruginosa* including first, it can grow and multiply in liquid food, second, it can form biofilm on the surface of food processing pipes or containers, and third, it can form biofilm on the surface of solid food. For the IPL irradiation conditions, three different capacitances (650 μF, 470 μF, and 220 μF) were set and the flux received by microbial cells was controlled by setting different pulse frequencies, and then the inactivation rate was calculated by colony culture and counting to determine the inactivation effect of IPL on *P. aeruginosa* in different growth states.

### 2.2. Strain and its activation methods

*Pseudomonas aeruginosa* ATCC27853 was used in this study and the strain was stored in our laboratory in a −80°C freezer. To activate the strain, an inoculating loop was dipped into the melted *P. aeruginosa* solution and then scribed on a TSA plate. The plate was placed in a 37°C incubator and incubated for 16–20 h. A single colony was picked and transferred to 1.5 mL of TSA medium and incubated in a shaker at 37°C for 12 h at 200 rpm. To obtain the *P. aeruginosa* ATCC27853 solution that can be used for subsequent experiments, 100 μL of the overnight culture was transferred to 3 mL of freshly prepared TSB medium and incubated at 37°C, 200 rpm, for 4 h.

### 2.3. Growth conditions

1. Tryptone Soy Broth (TSB) Medium [9.0 g TSB powder (Huankai Guangzhou, China) dissolved in 300 mL of distilled water, autoclaved at 121°C, 101 KPa for 15 min] was used for liquid culture of *P. aeruginosa* ATCC27853.
2. Tryptone Soy Agar (TSA) Medium [add 1.5% (w/v) agar powder to TSB, autoclaved at 121°C, 101 KPa for 15 min] was used for colony count of *P. aeruginosa* ATCC27853.
3. 0.9% saline: 5.4 g of sodium chloride was added to 600 mL of distilled water, stirred well until completely dissolved, placed in a sterilizer and set at 121°C, 101 KPa and autoclaved for 15 min.

### 2.4. Planktonic state modelling

The bacterial solution in 2.1 was diluted to 10^6^ CFU/mL with TSB medium, 100 μL of the dilution was coated on a TSA plate, and then the plate with dried surface was irradiated with IPL of three different capacitances and corresponding different irradiation frequencies. The control group was not irradiated with IPL. Finally, the irradiated experimental and control plates were placed in an incubator at 37°C and the colonies were counted after 24 h of incubation. Three parallel tests for each sample.

### 2.5. 96-well cell plate biofilm modeling

*Pseudomonas aeruginosa* ATCC27853 solution at a concentration of approximately 10^5^ CFU/mL was added to 96-well cell plates at 200 μL per well and incubated at 37°C for up to 8 h (early biofilm) or 48 h (mature biofilm), changing the fresh TSB medium every 12 h. After incubation, the supernatant was removed and the biofilm was washed three times with 200 μL saline. The 96-well cell plate with biofilm attached was then placed in an IPL device and sterilized with three different capacitances and different irradiation frequencies. After treatment with IPL, the biofilm was blown with 200 μL of saline to collect *P. aeruginosa* in the biofilm and repeated three times. A total of 600 μL of the collected *P. aeruginosa* solution was diluted in a gradient and spread on TSA plates at 37°C and incubated for 24 h before counting. Set up three parallel experiments.

### 2.6. Polycarbonate membrane modeling

The polycarbonate membrane was irradiated with UV light for 30 min on both sides before use. Ten microliter of *P. aeruginosa* solution at a concentration of approximately 10^5^ CFU/mL was transferred onto a polycarbonate membrane and incubated at 37°C for 8 h (early biofilm) or 48 h (mature biofilm). The polycarbonate membrane with biofilm attached was then placed in an IPL device and sterilized with three different capacitances and different irradiation frequencies. After treatment with IPL, the polycarbonate membrane was put into a tube containing 8 mL of saline and sonicated for 10 min using an ultrasonic device Saline containing *P. aeruginosa* was diluted in a gradient and coated onto TSA plates at 37°C and incubated for 24 h before counting. Set up three parallel experiments.

### 2.7. IPL parameters

For the treatment of planktonic *P. aeruginosa*, plates were placed 15 cm directly below xenon lamp and irradiated for 15, 30, and 45 pulses, 15, 30, and 45 pulses, and 30, 45, and 60 pulses with the capacitances of 650 μF, 470 μF and 220 μF, respectively. For the treatment of *P. aeruginosa* biofilm in the 96 well cell culture plate, plates were placed 15 cm directly below xenon lamp and irradiated for 60, 180, and 360 pulses, 180, 360, and 540 pulses, and 360, 540, and 720 pulses with the capacitances of 650 μF, 470 μF and 220 μF, respectively. For the treatment of *P. aeruginosa* biofilm in the polycarbonate membrane, plates were placed 15 cm directly below xenon lamp and irradiated for 180, 360, and 540 pulses, 540, 720, and 900 pulses, and 900, 1,080, and 1,260 pulses with the capacitances of 650 μF, 470 μF and 220 μF, respectively.

### 2.8. Statistical analysis methods

The statistical analysis was conducted using the SPSS software (version 26.0) with the One-way analysis of variance (ANOVA) test to determine significant differences between the experimental groups and the control group. The diagrams were drawn using the software GraphPad Prism 5. *p* value of ≤0.05 was considered statistically different and was marked as “*.” *p* value of ≤0.01 was considered statistically significantly different and was marked as “**.” *p* value of ≤0.001 was considered extremely statistically significantly different and was marked as “***.”

## 3. Results

### 3.1. Sterilization effect of IPL on *Pseudomonas aeruginosa* in water and liquid foods

**Figure 2:**
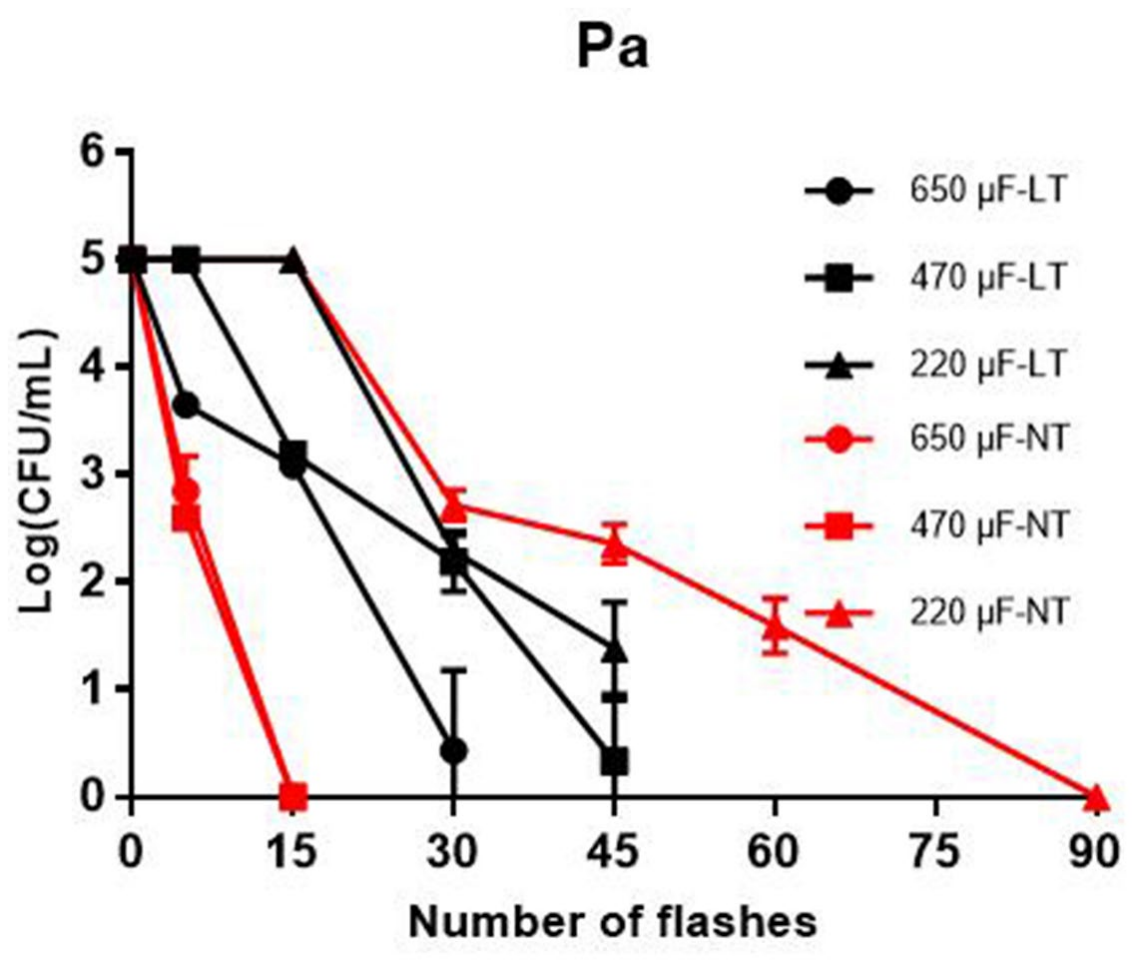
Reduction in cultivable number of *P. aeruginosa* in planktonic cultures. The IPL device is set with three different capacitance parameters, 650 μF, 470 μF, and 220 μF. LT means low temperature (4°C) and NT means normal temperature (25°C).

As a Gram-negative pathogen, *P. aeruginosa* is often detected in liquid foods such as drinking water, milk and fruit juices. To investigate the bactericidal effect of IPL on *P. aeruginosa* in liquid food, we simulated *P. aeruginosa* in liquid food with *P. aeruginosa* in the planktonic state and then tested the bactericidal effect of IPL on *P. aeruginosa* in the planktonic state. The results showed that at 25°C, with a capacitance of 650 μF and 480 μF, sterilization rates of over 99% can be achieved at 15 irradiation frequencies. And at 30 and 45 irradiation frequencies, the germicidal rate can reach 100%. With a capacitance of 220 μF, sterilization rates of over 99% can be achieved at 30 irradiation frequencies. And at 45 and 60 irradiation frequencies, the germicidal rate can reach over 99.9%. At 4°C, with a capacitance of 650 μF, 480 μF and 220 μF, sterilization rates of over 99.9% can be achieved at 30, 30, and 45 irradiation frequencies, respectively (Figure 2 and Table 1).

**Table 1:** Inactivation rate of IPL on planktonic cultures.

Strain	Temperature (°C)	Capacitance					
		650 μF		470 μF		220 μF	
		The number of irradiations	Rate of inactivation (%)	The number of irradiations	Rate of inactivation (%)	The number of irradiations	Rate of inactivation (%)
*P. aeruginosa*	25	15	99.14	15	99.60	30	99.76
		30	100.00	30	100.00	45	99.96
		45	100.00	45	100.00	60	100.00
	4	15	98.75	15	98.20	30	99.80
		30	99.99	30	99.97	45	99.97
		45	100.00	45	100.00	60	100.00

### 3.2. Sterilization effect of IPL on biofilm formed by *Pseudomonas aeruginosa* on 96-well cell plate biofilm modeling

**Figure 3:**
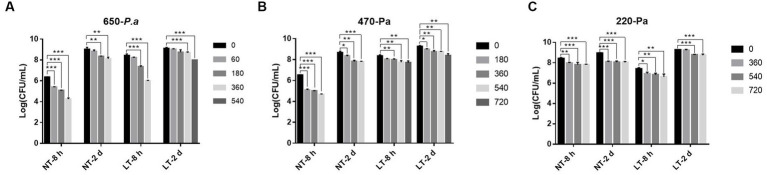
IPL sterilization efficiency on biofilm of *P. aeruginosa* in the 96-well cell culture plate model. **(A)** Bactericidal effect of IPL at a capacitance of 650 μF on *P. aeruginosa*. **(B)** Bactericidal effect of IPL at a capacitance of 470 μF on *P. aeruginosa*. **(C)** Bactericidal effect of IPL at a capacitance of 220 μF on *P. aeruginosa*. The asterisks denote statistical significance as determined by Tukey’s mean comparison test (“^***^” means *p* < 0.001, “^**^” means 0.001 < *p* < 0.01, “^*^” means 0.01 < *p* < 0.05). Error bars indicate standard deviation from three independent experiments.

*Pseudomonas aeruginosa* has a strong biofilm forming ability and can form biofilms on surfaces of biotic or abiotic materials, including milk transfer lines, surfaces of nutrient laden containers, etc. To investigate the bactericidal ability of IPL on biofilm formed by *P. aeruginosa* on the surface of a container, biofilm formed on a 96-well cell plate was used to simulate biofilm on the inner wall of a pipe or on the surface of a container, and the bactericidal effect of IPL on the bacteria in the biofilm was tested. As can be seen in Figure 3 and Supplementary Table S1, at 25°C, with a capacitance of 650 μF and 180 irradiation times, the bactericidal rate of early biofilm can reach 94.39%, and with 360 irradiation times, the bactericidal rate of early biofilm can reach 99.17%. However, with a capacitance of 650 μF and 360 irradiation times, the bactericidal rate for mature biofilm was still below 90%, at 88.24%. With a capacitance of 470 μF, the sterilization rate of *P. aeruginosa* early biofilm was above 95% at three different irradiation times of 180, 360 and 540, with a sterilization rate of 96.35%, 97.02% and 98.65%, respectively. For the same IPL parameters, the sterilization rate for *P. aeruginosa* mature biofilm was below 90%, with 53.57%, 85.71% and 86.43%, respectively. With a capacitance of 220 μF, the sterilization rate for *P. aeruginosa* early biofilm was above 85% and below 90% at three different irradiation times of 360, 540, and 720. For the same IPL parameters, the sterilization rate for *P. aeruginosa* mature biofilm was below 75%. Based on Supplementary Table S1 and Figure 3, it can be seen that with a capacitance of 470 μF and irradiation frequencies of 180, 360, and 540, the bactericidal rate of IPL on early biofilms at 4°C was significantly lower compared to 25°C. However, under other capacitance conditions, there was no significant difference in the bactericidal effect of IPL at 4°C and 25°C.

### 3.3. Sterilization effect of IPL on biofilm formed by *Pseudomonas aeruginosa* on polycarbonate membrane modeling

**Figure 4:**
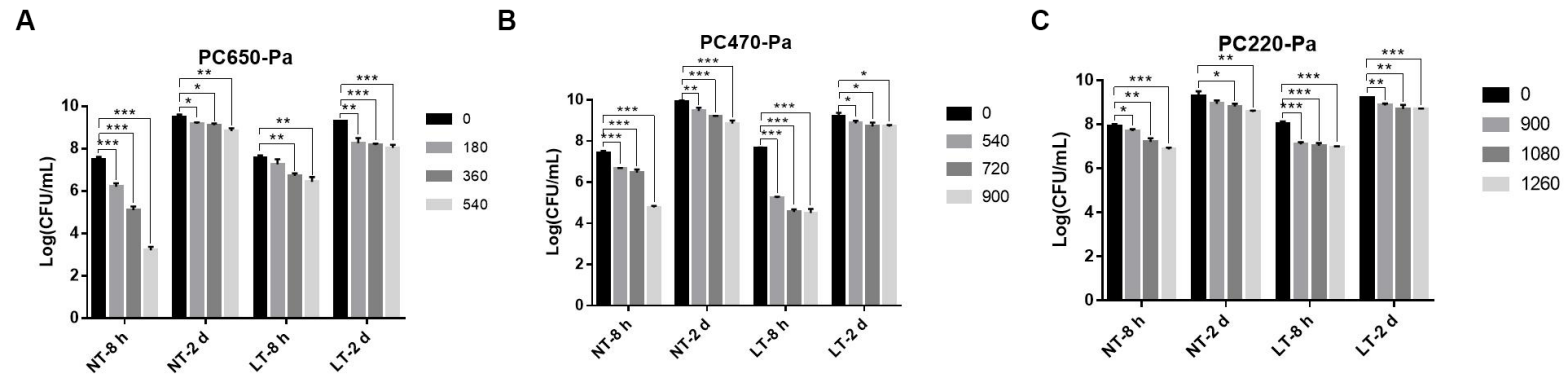
IPL sterilization efficiency on biofilm of *P. aeruginosa* in polycarbonate membrane model. **(A)** Bactericidal effect of IPL at a capacitance of 650 μF on *P. aeruginosa*. **(B)** Bactericidal effect of IPL at a capacitance of 470 μF on *P. aeruginosa*. **(C)** Bactericidal effect of IPL at a capacitance of 220 μF on *P. aeruginosa*. The asterisks denote statistical significance as determined by Tukey’s mean comparison test (“^***^” means *p*   <   0.001, “^**^” means 0.001  < *p*     <   0.01, “^*^” means 0.01 <  *p* < 0.05). Error bars indicate standard deviation from three independent experiments.

In addition to forming biofilm on the surface of vessels, *P. aeruginosa* is also capable of forming biofilm on the surface of food that is exposed to air. To investigate the bactericidal effect of IPL on the biofilm of *P. aeruginosa* on food surfaces, we simulated the biofilm of *P. aeruginosa* on food surfaces with biofilm formed on polycarbonate membranes. As can be seen from Figure 4, the efficiency of the IPL in sterilizing the *P. aeruginosa* early biofilm at 650 μF capacitance and three different irradiation frequencies at 25°C showed an extremely significant difference (*p* < 0.001) compared to the control group, and the sterilization rate for the mature film was statistically difference (*p* < 0.01). The bactericidal effect of IPL on *P. aeruginosa* early biofilm at 470 μF capacitance at 4°C and 25°C and at three different irradiation frequencies of 540, 720, and 900 was highly significant compared to the control, with a bactericidal rate of >80% (*p* < 0.001). With the same IPL parameters, the sterilization effect of IPL on mature *P. aeruginosa* biofilm is higher at 25°C than at 4°C. For example, with a capacitor of 470 μF and an irradiation frequency of 900, the sterilization rate is 91.2% at 25°C (*p* < 0.001), compared to 70% at 4°C (*p* < 0.05), Supplementary Table S1. The bactericidal rate of IPL against early *P. aeruginosa* biofilm was >90% at two different temperatures with a capacitance of 220 and a frequency of 1,260 irradiations times (*p* < 0.001). With a capacitance of 220 μF, the bactericidal effect of IPL on *P. aeruginosa* mature biofilm was below 80% at 25°C and 4°C at three different irradiation frequencies of 900, 1,080 and 1,260, except at 25°C where the bactericidal rate was 81.67% at an irradiation frequency of 1,260 (*p* < 0.05).

## 4. Discussion

The IPL was successfully applied to sterilize *P. aeruginosa* in the planktonic state and on two different models, specifically, the highest bactericidal rates for the planktonic state and early biofilm on two models of 96-well plates and polycarbonate membranes was of 100%, 99.63%, and 99.99%, respectively. Our results is consistent with the findings of Yi et al. (2017), who found that IPL inactivation curves of *P. aeruginosa* varies with pulse conditions, and the higher the capacitance for a given irradiation frequency, the higher the mortality of *P. aeruginosa*. And when the capacitance conditions are constant, the higher the frequency of irradiation, the higher the sterilization rate. However, the drawback is that we did not investigate whether there is a linear relationship between irradiation frequency and bactericidal rate, and only tested the bactericidal effect on *P. aeruginosa* at three different irradiation frequencies selected for each capacitance condition based on the results of previous studies (Liu et al., 2021).

In order to investigate whether IPL can sterilize *P. aeruginosa* on the surface of food in a refrigerator or cold storage room, we tested the sterilization rate of *P. aeruginosa* by IPL at 25°C and 4°C. The results show that temperature had an effect (*p* < 0.05) on the sterilization of early biofilm formed on 96-well cell plates when treated with a 470 μF capacitor, with better results at 25°C. However, when the biofilm formed on polycarbonate membranes was treated with 650 μF capacitance (*p* < 0.05), the sterilization effect was better at 4°C. Although temperature (4°C and 25°C) has been observed to impact the efficacy of IPL in killing *P. aeruginosa* under certain conditions, further research is needed to determine the generality of this effect. This result is inconsistent with our previous finding that temperature had no significant effect on bactericidal efficiency when IPL treated seven different food-borne Gram-positive bacteria and molds in food (Li et al., 2023a,b). Chen et al. (2018, 2020) found the best inactivation of *Cronobacter sakazakii* by IPL was achieved synergistically with a water activity of 0.25 and a residence time of 28 s with IPL at a temperature of approximately 57°C.

Based on the data results in Figures 3, 4 and Supplementary Tables S1, S2, it can be seen that the maturity of biofilm affects the bactericidal effect of IPL on *P. aeruginosa*, with mature biofilm at 48 h being more difficult to kill than early biofilm. The biofilm matrix consisting of polysaccharides, proteins and eDNA acts as a protective barrier to protect the bacteria in the biofilm against UV, antibiotics and other hostile environments (Bakke et al., 1984; Mah et al., 2003; Driffield et al., 2008). The study of Mehrangiz Ghasemi and colleagues showed that the combination of curcumin and silver nanoparticles could hinder *P. aeruginosa* planktonic and biofilm formation and also demonstrated that curcumin was effective in killing planktonic forms of bacteria and that its anti-biofilm effect was limited (Ghasemi et al., 2021). Martinez et al. (2020) also concluded that disrupting and eradicating the biofilm of highly relevant pathogenic bacterial species poses a greater challenge than removing free cells. Shih and Lin (2010) suggested that pathogens consistently resident in biofilms are significantly more resilient to antiseptics than planktonic cells. Finding ways to control pathogens in biofilms is a more important and urgent matter.

From the results of this study, it can be seen that *P. aeruginosa* in the free state on PDA plates is most susceptible to IPL treatment, followed by biofilm formed by *P. aeruginosa* on 96-well cell plates and finally by *P. aeruginosa* on polycarbonate membranes. The three models above simulate floating *P. aeruginosa* in liquid, *P. aeruginosa* biofilm on the walls of food transport pipes or containers, and *P. aeruginosa* biofilm formed on the surface of food, respectively. We consider that the presence of *P. aeruginosa* in the food significantly affects the effectiveness of IPL, however, a serious drawback of IPL sterilization is that it can only be used to sterilize the surface of food or food processing equipment, devices and not the microorganisms hidden inside the food. However, IPL has the advantage that it has little effect on the flavor and nutritional content of foods and can be used to extend the shelf life of foods packaged in transparent materials and fresh foods. The range of pulsed intense light that acts as a germicidal agent is probably UV, but other ranges may have synergistic effects. In combination with the type of food being processed and the characteristics of each sterilization method, different sterilization methods should be selected for the food processing process, such as ultra-high-pressure sterilization, low temperature sterilization, ultra-high temperature instant sterilization, microwave sterilization, ultraviolet sterilization, far infrared sterilization, etc.

## 5. Conclusion

This study tested the effectiveness of IPL in inactivation of *P. aeruginosa* on three different models. In addition, the effects of capacitance, number of pulses, temperature and biofilm maturity on the bactericidal effect of IPL were also tested. The results revealed that the bactericidal effect of IPL on *P. aeruginosa* was significantly influenced by the state of the bacterium, the maturity of the biofilm and the material to which *P. aeruginosa* was attached. Compared to *P. aeruginosa* in the planktonic state, IPL showed significantly lower inactivation of *P. aeruginosa* in the biofilm state, and the more mature the biofilm, the lower the bactericidal efficiency. Under the same capacitance conditions, for the same state of growth of *P. aeruginosa*, the higher the number of pulses, the better the bactericidal effect. In addition, the IPL showed significantly lower inactivation of biofilm on polycarbonate membranes compared to biofilm on 96-well cell plates. Overall, IPL was effective in killing *P. aeruginosa* in the planktonic state modelling, 96-well cell plate biofilm modeling, and polycarbonate membrane modeling, making IPL a promising sterilization method.

## Data availability statement

The original contributions presented in the study are included in the article/Supplementary material, further inquiries can be directed to the corresponding authors.

## Author contributions

XL and YG conceived of the study and participated in its design and coordination. JL and TH performed the experimental work and analyzed the data. JL and YG prepared and revised this manuscript. All authors contributed to the article and approved the submitted version.

## Funding

This work was supported by the Projects of Enterprise Sci-tech Commissoner of Guangdong (No. GDKTP2021036400), the Projects of Science and Technology of Yunfu (No. 2022010220), and the 111 Project (B17018).

## Conflict of interest

The authors declare that the research was conducted in the absence of any commercial or financial relationships that could be construed as a potential conflict of interest.

## Publisher’s note

All claims expressed in this article are solely those of the authors and do not necessarily represent those of their affiliated organizations, or those of the publisher, the editors and the reviewers. Any product that may be evaluated in this article, or claim that may be made by its manufacturer, is not guaranteed or endorsed by the publisher.
