# Supplementary material for: Germicidal effect of intense pulsed light on Pseudomonas aeruginosa in food processing
Source: Front Microbiol. 2023 Aug 24;14:1247364. doi: 10.3389/fmicb.2023.1247364 (PMC10484712; doi:10.3389/fmicb.2023.1247364)
Supplement: Supplementary file 1 [file Table_1.DOCX]

**Supplementary Materials**

Table S1. Inactivation rate of IPL on biofilm in the 96 well cell culture plate model.

| Strain | Temperature | Capacitance | | | | | | | | | | | |
| --- | --- | --- | --- | --- | --- | --- | --- | --- | --- | --- | --- | --- | --- |
|  |  | 650 μF | | | | 470 μF | | | | 220 μF | | | |
|  |  | 8h | | 2d | | 8h | | 2d | | 8h | | 2d | |
|  |  | the number of irradiations | Rate of inactivation | the number of irradiations | Rate of inactivation | the number of irradiations | Rate of inactivation | the number of irradiations | Rate of inactivation | the number of irradiations | Rate of inactivation | the number of irradiations | Rate of inactivation |
| P. aeruginosa | 25 ℃ | 60 | 88.48% | 60 | 38.24% | 180 | 96.35% | 180 | 53.57% | 360 | 86.30% | 360 | 63.75% |
|  |  | 180 | 94.39% | 180 | 80.59% | 360 | 97.02% | 360 | 85.71% | 540 | 86.67% | 540 | 72.50% |
|  |  | 360 | 99.17% | 360 | 88.24% | 540 | 98.65% | 540 | 86.43% | 720 | 87.41% | 720 | 75.00% |
|  | 4 ℃ | 60 | 38.75% | 60 | 51.35% | 180 | 57.14% | 180 | 68.52% | 360 | 73.75% | 360 | 68.33% |
|  |  | 180 | 91.63% | 180 | 59.46% | 360 | 74.29% | 360 | 68.52% | 540 | 82.50% | 540 | 71.67% |
|  |  | 360 | 99.63% | 360 | 91.89% | 540 | 77.14% | 540 | 85.19% | 720 | 82.22% | 720 | 78.33% |

Table S2. Inactivation rate of IPL on biofilm in the polycarbonate membrane model.

| Strain | Temperature | Capacitance | | | | | | | | | | | |
| --- | --- | --- | --- | --- | --- | --- | --- | --- | --- | --- | --- | --- | --- |
|  |  | 650 μF | | | | 470 μF | | | | 220 μF | | | |
|  |  | 8h | | 2d | | 8h | | 2d | | 8h | | 2d | |
|  |  | the number of irradiations | Rate of inactivation | the number of irradiations | Rate of inactivation | the number of irradiations | Rate of inactivation | the number of irradiations | Rate of inactivation | the number of irradiations | Rate of inactivation | the number of irradiations | Rate of inactivation |
| P. aeruginosa | 25 ℃ | 180 | 94.44% | 180 | 48.89% | 540 | 83.75% | 540 | 64.00% | 900 | 42.31% | 900 | 55.00% |
|  |  | 360 | 99.56% | 360 | 56.67% | 720 | 88.75% | 720 | 81.60% | 1080 | 80.77% | 1080 | 66.67% |
|  |  | 540 | 99.99% | 540 | 76.67% | 900 | 99.78% | 900 | 91.20% | 1260 | 91.54% | 1260 | 81.67% |
|  | 4 ℃ | 180 | 45.45% | 180 | 90.00% | 540 | 99.62% | 540 | 56.00% | 900 | 88.48% | 900 | 53.19% |
|  |  | 360 | 85.45% | 360 | 92.67% | 720 | 99.92% | 720 | 68.00% | 1080 | 90.00% | 1080 | 65.96% |
|  |  | 540 | 91.82% | 540 | 94.33% | 900 | 99.92% | 900 | 70.00% | 1260 | 91.82% | 1260 | 70.21% |
